# A pharmaceutical policy accident: collision of shareholder capitalism and Chinese state capitalism driving the shortage of an essential antibiotic

**DOI:** 10.1080/20523211.2024.2430441

**Published:** 2024-12-03

**Authors:** Nadya Wells, Vinh-Kim Nguyen, Stephan Harbarth

**Affiliations:** a Global Health Centre, Geneva Graduate Institute, Geneva, Switzerland; b Faculty of Medicine, University of Geneva, Geneva, Switzerland; c Department of Anthropology, Geneva Graduate Institute, Geneva, Switzerland; d Infection Control Program, University of Geneva Hospitals and Faculty of Medicine, WHO Collaborating Center, Geneva, Switzerland

**Keywords:** Antibiotic shortages, piperacillin-tazobactam, API, pharmaceutical policy, AMR, supply chain, resilience, global health security, capitalism, China, Pfizer

## Abstract

**Background:**

An explosion in a Chinese factory in 2016 caused a global shortage of essential broad-spectrum antibiotic piperacillin-tazobactam. Hitherto, no detailed, policy-relevant analysis has been conducted on this major shortage event. Thus, we aimed to (1) investigate causes; (2) describe supply chain challenges; and (3) uncover policy gaps to support possible mitigation actions.

**Methods:**

Applying an analytical framework for security of medical supply chains, we investigated the changing roles of Pfizer-led and Chinese API suppliers. We identified demand surge, capacity reduction and co-ordination failures. Triangulating between scientific literature, corporate, and regulatory documents, we analysed the impact of Western and Chinese policy contexts on supply chain resilience.

**Results:**

We uncovered ‘red flags’: geographically dispersed manufacturing failures due to complexity of sterile production; undetected supply chain concentration and interlinkages; and Chinese policy-led API supplier consolidation. We found these warning signals were ignored in the absence of a co-ordinated policy framework to identify and mitigate emerging global supply risks. Firstly, policy makers lacked visibility on growing ‘volume dependency’ in the chain. Secondly, national policy makers lacked a global view of supply risk. Thirdly, we show antibiotic API manufacturing economics were impacted by a number of non-pharmaceutical policy decisions (e.g. state aid, environmental standards, procurement rules) which contributed to supply chain vulnerability.

**Conclusions:**

Our findings suggest possible policy gaps in governance of supply chain resilience. Firstly, disclosure of API suppliers including degree of dependency may better pre-empt bottlenecks, facilitating priority setting for public investments in re-shoring where global API supply currently relies on few, or single plants; secondly, a whole-of-government approach may counter the potential impact of non-pharmaceutical policies on supply chain resilience. Our findings confirm suggestions from previous studies that international data sharing would be beneficial considering the global shortage effects which can emerge from a single point of failure.

## Background

In 2016, an explosion in a Chinese factory generated a global shortage of piperacillin-tazobactam (pip-tazo), an essential broad-spectrum antibiotic agent (Skender & Zhang, 2024). The global pip-tazo supply chain collapsed because it had become reliant on a single supplier (Oehler & Gompf, 2020). At that moment in time, a key active pharmaceutical ingredient (API) in pip-tazo was only produced in that single factory (Access to Medicines Foundation, 2018). The antibiotic, a derivative of penicillin, is widely used in critical care because of its broad spectrum of coverage (Perry & Markham, 1999). Clinical trials have demonstrated safety and tolerability of pip-tazo for the treatment of abdominal, skin, and lower respiratory tract infections (Gin et al., 2007). Indeed, pip-tazo is considered as the most essential antimicrobial agent for treating most nosocomial infections, including hospital-acquired pneumonia (Ghadimi et al., 2024). The shortage therefore had a significant clinical impact, requiring hospitals worldwide to move to alternative treatments (Barber et al., 2016; Cunha, 2016; Gross et al., 2017; Kessel et al., 2018; King et al., 2017). Risks to antimicrobial stewardship programmes emerged due to pip-tazo’s widespread use in treatment of polymicrobial infections (Sanders & Sanders, 1996).

A recent historical study demonstrated that antibiotic shortages have grown since the 1970s from temporary local incidents to a global concern affecting clinical care (Skender & Zhang, 2024). Antibiotic shortages have been shown to hamper effective treatment of bacterial infections (Griffith et al., 2012; Harbarth, 2000; Quadri et al., 2015). Suboptimal treatment regimens contribute to antimicrobial resistance, a public health emergency threatening the foundations of modern medicine (Tängdén et al., 2018; WHO, 2014). Antibiotic research and development faces a series of scientific, regulatory and economic challenges leading to a lack of access to novel agents to counter emerging multiresistant infections (Årdal et al., 2020; Outterson et al., 2021; Vagneron et al., 2024; Wells et al., 2024). Moreover, shortages of older generic antibiotics, have been identified as a critical but neglected priority (Pulcini et al., 2017). Recognised causes include low financial returns, limited markets, and insufficiently diversified supply chains (WHO, 2018).

Global supply chains became increasingly concentrated as Western pharmaceutical companies closed antibiotic production in Europe and the US, outsourcing to lower cost manufacturers, primarily in Asia (Oehler & Gompf, 2020). Particularly since the COVID-19 pandemic which highlighted the supply risks emanating from geographically concentrated manufacturing (Guerin et al., 2020), there has been a significant renewal in attention towards supply chain resilience (SCR) (Faggioni et al., 2024), with a noticeable increase in studies published on the pharmaceutical sector (Faggioni et al., 2023; Gupta & Kayande, 2023). In the quest for novel approaches to building resilience and frameworks for ‘key resilience attributes’, Faggioni et al have highlighted the importance of synchronisation between parties within the supply chain, a capability to identify and mitigate risks, and the increased vulnerabilities that emerge from global footprints.

However, knowledge gaps have hindered attempts to re-build SCR (Katsaliaki et al., 2022), leaving medicine supply vulnerable to shocks. While scholars have established definitions for SCR, they have also pointed out the gap in understanding ‘how a supply chain could practically be resilient to disruptions’ (Gupta & Kayande, 2023). In a major review of supply chain disruptions and resilience, Katsaliaki et al point out that empirical evidence on the effects of supply chain disruptions is limited (Katsaliaki et al., 2022). Considering this SCR research agenda, which seeks to understand specific causes and potential mitigations, the pip-tazo shortage presents an important opportunity for an empirical case study due to its global and prolonged impact. Indeed, a recently published systematic review of global antibiotic shortages (2000–2023) demonstrated that shortages of pip-tazo were those most reported (28.4%) over the 23-year period (Pandey et al., 2024); yet hitherto, no detailed policy-relevant analysis has been conducted on this major shortage event.

While in some jurisdictions, national regulatory authorities ask market authorisation holders to name their API suppliers, there is typically neither obligatory disclosure of degree of dependency on specific companies or plants (Lynn, 2020), which in this paper we characterise as ‘volume dependency’, nor systematic information exchange between countries (Årdal et al., 2021). The absence of co-ordinated oversight from policy makers means there is no early warning mechanism when global supply chains become dependent on one API producer. In this retrospective study, we tracked pip-tazo’s API supply chain focussing on events before and after the 2016 collapse. We aimed to (1) investigate causes; (2) describe supply chain challenges; and (3) uncover policy gaps to support possible mitigation actions. We show that, rather than being a ‘one-off’, extraordinary event, the sudden collapse of pip-tazo supply worldwide is symptomatic of how global supply chains for antibiotic production have become vulnerable to single points of failure (Pandey et al., 2024). This supply chain fragility is a major global health risk (WHO, 2023).

## Methods

Firstly, we established a shortage timeline. US shortage data was chosen because it provides a comprehensive, consistently defined, and long-term data set as described below. This showed a new series of shortages beginning in 2010, only resolved in 2020. We therefore took Pfizer’s acquisition of piperacillin-tazobactam in 2009 as our starting point (Pfizer Inc, 2010). Knowing that the collapse happened due to reliance on one factory in China by 2016, we then juxtaposed the emerging role of Chinese API suppliers, referring to WHO analysis of Chinese policies to promote local production (WHO, 2017). We sought to identify signals of increasing supply chain risk, which we interpret as warning signs and characterise in this paper as ‘red flags’.

### Data sources

Comprehensive long-term antibiotic shortage data is available from an extensive US dataset compiled by the University of Utah, using previously published methods (Fox & Tyler, 2003). UUDIS define a shortage as ‘a supply issue that affects how the pharmacy prepares or dispenses a drug product or influences patient care when prescribers must use an alternative agent’ (Mazer-Amirshahi et al., 2017). We generated a focussed dataset covering US pip-tazo shortages from 2000 to 2020. This allowed us to constitute a shortage timeline.

We then sought to identify capacity constraints emerging in Pfizer-led and Chinese API supply chains within this timeline. We analysed Pfizer’s changing role in the supply chain drawing on scientific literature, corporate, policy and regulatory documents. We did this by, firstly, building a financial dataset using Pfizer annual reports (2005–2018), and shareholder proxy statements (2011–2018). This allowed us to identify corporate actions which impacted API supply: merger (Wyeth, 2009), acquisition (Hospira, 2015) and joint venture (Hisun, 2012–2017). Secondly, broader electronic searches were undertaken in Google in December 2023, using search terms ‘piperacillin-tazobactam’ and firm names: ‘Wyeth’ (112 results), ‘Hospira’ (98 results), ‘Hisun’ (15 results). We identified policy documents, government-sponsored shortage and reshoring studies, regulatory interventions, and competition enquiries^1^ related to pip-tazo. These were manually searched to identify emerging capacity constraints and policy maker reactions. The clinical impact of the shortage during the study period was examined in scientific literature (PubMed search term ‘piperacillin tazobactam shortage’ including articles describing shortages 2010–2020 which generated 16 studies). Finally, we consulted the WHO, 2017 report on China Policies to promote local production (WHO, 2017) in order to trace the evolving policy environment for Chinese API producers.

#### Data limitations and mitigation

The pharmaceutical industry often keeps API production locations a commercial secret contributing to knowledge gaps for scholars. Private firms see commercial advantage in keeping their supply locations secret for several reasons. Firstly, disclosure could alert competitors to supply chain weaknesses. Secondly, production costs could be calculated using supplier financial data. Thirdly, health system procurement processes are often run on tenders which privilege the lowest price; transparency therefore could enable manufacturers to use financial information to drive out competition (Årdal et al., 2021). Even the 2023 French government antibiotic shortage study relied on ‘a goodwill basis’ to collect information on production sites from the industry (WHO, 2023). Plant level data is often hidden from the public, either behind commercial agreements or within private datasets and only shared between market authorisation holders and their national regulators (Årdal et al., 2021). This opacity is a key limitation of studies of API supply chains, including ours (Grau Granada & Wanner, 2019).

Three approaches were used to mitigate these methodological limitations. Firstly, we do not claim to investigate every potential supplier of pip-tazo. Acknowledging inherent opacity, we focussed our study on investigating the interactions of Pfizer’s changing role and the emerging role of Chinese suppliers of pip-tazo API. Secondly, knowing that firms do have obligations to disclose their suppliers to national or regional regulatory authorities in certain situations, and that these documents are sometimes made public, we sourced and compared regulatory data covering multiple jurisdictions (Europe, US, UK, China, India, New Zealand) in order to build a picture of policy interactions and attitudes to supply chain resilience during the time frame of our study. Thirdly, three experts were consulted (one UK research foundation, one Chinese researcher, one European private sector). These experts had knowledge of private datasets and were consulted to ensure our methodology enabled comprehensive coverage of pip-tazo’s API suppliers within the scope of our study.

### Supply chain analysis

**Figure 1.**
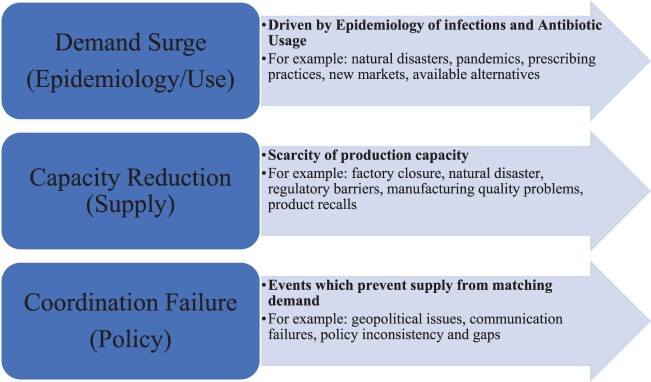
Framework of drivers of supply chain failure.

Supply chain management is a co-operative networked system achieving the flow of goods from supplier to end-consumer (Mentzer et al., 2001). For medical products, the supply chain often entails multiple geographically distinct entities. Our analytical framework is developed from the causes of medical supply chain failures identified by the US Committee on Security of America’s Medical Product Supply Chain (Shore et al., 2022). The committee determined that shortages can occur due to demand surge, capacity reduction or co-ordination failure. We incorporated these causes into a framework and presented our results and discussed our findings according to these categories (Figure 1).

## Results

### Demand surge

#### Piperacillin-tazobactam shortages in the US

Analysis of US shortage data shows the first reported ‘supply/demand’ mismatch began after Pfizer merged with Wyeth and thereby acquired piperacillin-tazobactam in 2009 (Table 1). The longest shortage, 2013–2020, has a reported ‘unknown’ cause. By mid-2015, seven US suppliers had their generics on back order. Fresenius Kabi USA, and Hospira reported consequently increased demand for their formulations which they could not fulfil. By November 2015, Sagent, Sandoz, and Mylan had formulations available, and Aurobindo Pharma was able to release smaller batches. Pfizer expected gradual recovery only into Q3 of 2017 (Pharmacy Learning Network, 2015). These results demonstrate that suppliers could not accommodate demand contributing to a major US shortage in 2015.

**Table 1.** Piperacillin-tazobactam and related drug shortages (2000–2020).

	Status 12.31.2022	Reason	Year	Date Notified	Date Resolved	Days Short
**Drug shortages**						
Piperacillin/tazobactam (Zosyn) injection	Resolved	Manufacturing problems	2001	05.03.01	12.03.0.	944
Piperacillin/tazobactam	Resolved	Manufacturing	2005	10.03.05	11.29.06	422
Piperacillin/tazobactam (Zosyn) injection	Resolved	Natural disaster	2005	24.01.05	02.08.05	190
Piperacillin/tazobactam	Resolved	Supply/demand	2010	07.13.10	05.11.11	302
Piperacillin/tazobactam injection	Resolved	Unknown	2013	05.08.13	09.22.20	2694
**Related drug shortages**						
Piperacillin (Pipracil)	Resolved	Manufacturing problems	2001	08.17.01	03.11.04	937
Piperacillin sodium	Resolved	Discontinued	2011	03.25.11	03.30.11	5
Ceftolozane/tazobactam vials (Zerbaxa)	Resolved	Manufacturing delay	2020	12.21.20	01.06.22	381

Source: University of Utah Drug Information Service (University of Utah Drug Information Service, 2024).

### Capacity reduction

#### Pfizer’s changing role

We found that unprecedented US shortages by December 2014 were caused by manufacturing issues at 2 of 8 suppliers, regulatory issues at another manufacturer, which combined with increased demand (Oehler & Gompf, 2020). Meanwhile, Pfizer’s supply constraints spilled over into 2016–2017. Analysing the supply side through competition enquiries, we found Pfizer’s acquisition of Hospira in 2015 exacerbated the shortage risk. While national regulators investigated potential market dominance, because the acquisition meant consolidation from two large global suppliers to one, we found that in fact Pfizer and Hospira were simultaneously experiencing capacity constraints. Financial data confirms Pfizer’s reduced role in global supply. Pfizer’s pip-tazo revenues under patent protection peaked at $952 m in 2010 falling to $303 m by 2014 with generic competition, subsequently being too small to be disclosed (Pfizer Inc, 2015). In Italy, Pfizer’s largest European market, regulators found market share had ‘drastically’ reduced from 60–70% in 2012 to 5–10% by 2014 (€70–80 m value) (European Commission, 2015). By 2016, Pfizer was struggling to supply API from Italy due to a delayed manufacturing upgrade (Gazzete Ufficiale, 2014). Meanwhile, Hospira’s beta-lactam plant in Irungattukottai, India suffered repeated manufacturing failures and was subject to FDA inspections and import bans from 2013 (FDA, 2018). A UK regulatory inspection in 2016 found the plant non-compliant with Good Manufacturing Practice leading to suspension of manufacturing (UK MHRA, 2016).

Meanwhile, in common with industry trends at the time, Pfizer sought to enhance profitability and cut costs through outsourcing. Analysis of our financial dataset showed Pfizer was under pressure to improve financial returns before the Wyeth merger due to multiple patent cliffs in high revenue products such as Zythromax (2005) and Lipitor (2006) (Pfizer Inc, 2005, 2006), poor R&D pipeline productivity (Pfizer Inc, 2008), and pricing pressure from medicine reimbursement reforms in high-income markets (Pfizer Inc, 2005). Since 2005, Pfizer has implemented a series of cost rationalisation plans in the face of falling revenues. These included closure of manufacturing plants (including API manufacturing in the UK in 2005 (Pfizer Inc, 2006)), downsizing of R&D sites and 49,000 job cuts in 2010 (Pfizer Inc, 2011).

Pfizer’s manufacturing rationalisation culminated in a Chinese joint venture. Hisun Pfizer Pharmaceuticals Company Limited, was established in 2012 to allow access to the Chinese market for Pfizer products and lower manufacturing costs for generics (Pfizer Inc, 2013). Hisun was a major pip-tazo API supplier, including both Pfizer and Hospira in 2015 (Changing Markets and Ecostorm, 2016). When Hisun was hit with a US FDA ban on imports due to data manipulation and manufacturing failures, the FDA noted consequent US shortage risk, which indeed materialised (Table 1) (FDA, 2015). Hisun had supplied 5.4 million doses in 2014, dropping to 180,000 in 2015, and 260,000 in 2016. Pfizer’s failure to compensate with API from Italy due to its own delayed manufacturing upgrade led to a $10 m compensation payment to Hisun in 2016 (Liu, 2017). Pfizer sold its stake in Hisun in 2017; thus, the joint venture did not deliver anticipated financial returns (Pfizer Inc, 2018).

#### Chinese supplier consolidation

In an additional ill-timed contribution to the supply chain’s eventual collapse, domestic policy changes impacted alternative Chinese suppliers. These encouraged the Chinese API industry to consolidate. Firstly, in 2015, the government introduced stricter rules on quality control and cleaner production, including ‘Prevention and Control of Water Pollution’, and an environmental protection tax, which pushed many API suppliers out of business, leading to an 18% reduction in the number of API manufacturers and medicine companies in China by 2016 (Fresenius Kabi, 2017). Secondly, domestic medicine purchasing moved to a system of tenders with quality criteria for APIs. This promoted economies of scale at companies that could conform and therefore again resulted in consolidation among suppliers (Nikkei Asia, 2022). Thirdly, policies to support self-sufficiency proved another driver of consolidation to ensure supply for the large domestic medicines market (WHO, 2017). These policies accelerated the emergence of API monopolies. The world became reliant on a shrinking number of Chinese companies, increasing the risk of capacity constraints.

#### The bottleneck and accident

Policy-driven supplier consolidation in China, Pfizer/Hospira’s constrained supply from Italy and India, and struggles at Pfizer/Hisun, left a Chinese company, Qilu Pharmaceuticals Co. Ltd. (Qilu), ‘the only facility producing the API needed’ (Access to Medicines Foundation, 2018). On October 10th, 2016, a pressure vessel containing ethanol in a workshop for waste water reclamation at Qilu exploded, halting the production of piperacillin (China Daily, 2016). The supply chain collapsed. The shortage had global impact (Barber et al., 2016; Cunha, 2016; Gross et al., 2017; King et al., 2017). It was so severe that the German Ministry of Health temporarily allowed treatments with unauthorised medicines (ECA, 2017) while British doctors described increased antibiotic resistance risks and higher hospital costs (Davis, 2017). This experience echoed the risks of adverse patient outcomes, and less effective and more costly treatment outlined in an earlier shortage survey among infectious disease specialists(Gundlapalli et al., 2013). A subsequent qualitative study conducted in 2016, aiming to contribute evidence to the methodological agenda of reporting and quantifying consequent patient harm(McLaughlin et al., 2014), called antimicrobial shortages ‘the new norm for infectious disease physicians’ with pip-tazo among the most commonly reported (Gundlapalli et al., 2018).

Indeed, the shortage provided an opportunity to analyse antimicrobial prescribing practices, the impact on hospital acquired infection rates (HAIs) and patient outcomes. A study in 88 US hospitals demonstrated greater hospital-onset *Clostridium difficile* (HO-CDI) rates because the shortage forced prescribers towards substitute antibiotics known to present higher HO-CDI risks(Gross et al., 2017)**.** A study in Brazil showed meropenem use increased 111% during the shortage with minimal impact on patients (measured in mortality rates and length of therapy) due to increased antimicrobial stewardship efforts (Barber et al., 2016). The variation in patient outcomes, HAI rates and prescribing practices in response to the shortage across different geographies reflected findings of studies in earlier pip-tazo shortage periods (Mendez et al., 2006). These reconfirmed the critical nature of antimicrobial stewardship programmes in addressing the impact on patient care (King et al., 2017; Urban et al., 2023) and their importance in reducing the effects of shortages as established in previous studies (Griffith et al., 2012).

#### The aftermath

Evidence from a UK parliamentary inquiry suggests Pfizer had supplies available again by July 2017 and would be able to bring in larger volumes by October. In 2018 Pfizer/Hospira’s Indian facilities were again constrained being mentioned as part of an FDA recall (The Economic Times, 2018), resulting in a shortage that was significant enough to elicit another question to the health minister on mitigations (Penning, 2018).

The shortage was reported resolved in 2020 (Table 1) but the supply chain into Europe still relied on a small number of suppliers (Table 2). Imports of piperacillin API were still dominated by two Chinese companies, Qilu and Shandong Ruiying Pharmaceutical Co. Ltd. Qilu was still the only company supplying the APIs of both piperacillin and tazobactam to EU importers. Only two Western companies, Fresenius Kabi and Istituto Biochimico Italiano Giovanni Lorenzini, were still registered as EU manufacturers of piperacillin and tazobactam. However, they both also imported API from China. By 2023, the French government antibiotic shortage study noted zero tazobactam manufacturing remaining in Europe (WHO, 2023), despite the introduction of a newer combination formulation involving tazobactam. Indeed, a shortage of ceftolozane/tazobactam was announced by the European Medicines Agency in December 2020, again due to contamination in manufacturing, which was only resolved in June 2022 (Table 1). In contrast, as of the end of 2020, there were more than 20 manufacturers in the piperacillin-tazobactam market in China, of which Pfizer Ltd had the largest market share (China Research and Inteligence Co., Ltd, 2021).

**Table 2.** EU piperacillin-tazobactam API import sources and destinations 2020.

Supplier country	API supplier	Import country	Importer
**Piperacillin**			
China	Qilu Pharmaceuticals Co. Ltd	Italy	Istituto Biochimico Italiano Giovanni Lorenzini S.P.A.
		Ireland	Mylan Teoranta
		Slovakia	Atb Pharma, S.R.O.
		Netherlands	Devrimed B.V., St.-Annaparochie
	Shandong Ruiying Pioneer Pharmaceutical Co. Ltd	Italy	Acs Dobfar S.P.A Fresenius Kabi Anti-Infectives S.R.L.
			Fresenius Kabi Ipsum S.R.L.
		Belgium	Pfizer Service Company Bvba
Korea	Yuhan Chemical Inc	Belgium	Pfizer Service Company Bvba
India	Mangalam Drugs And Organics Ltd	Italy	Afasigma S.P.A.
**Tazobactam**			
China	Qilu Pharmaceuticals Co. Ltd	Italy	Acs Dobfar S.P.A.
		Netherlands	Devrimed B.V., St.-Annaparochie
	Shandong Anxin Pioneer Pharmaceutical Co. Ltd	Italy	Emgi Srl
	Zhejiang Hualbang Medical & Chemical Co. Ltd	Italy	Fresenius Kabi Anti-Infectives S.R.L.
			Fresenius Kabi Ipsum S.R.L.
	Jiangxi Fushine Pharmaceutical Co. Ltd	Italy	Fresenius Kabi Anti-Infectives S.R.L.
			Fresenius Kabi Ipsum S.R.L.
Japan	Otsuka Chemical Co. Ltd	Italy	Acs Dobfar S.P.A.
			Fresenius Kabi Ipsum S.R.L.

Source: EudraGMDP.

## Discussion

**Figure 2.**
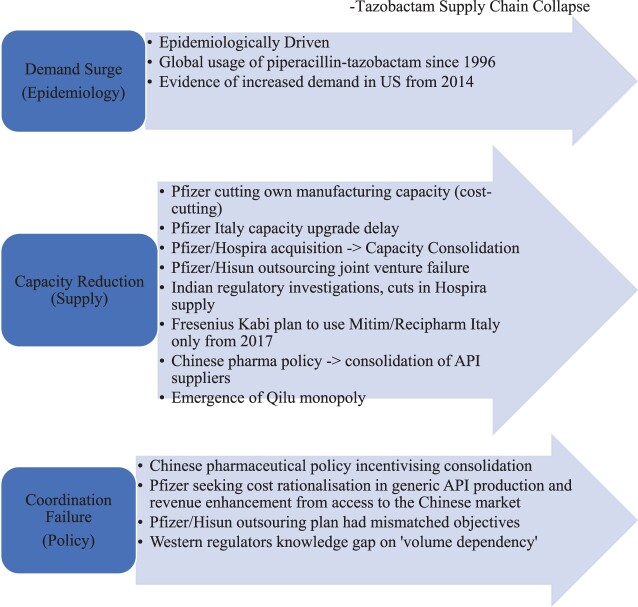
Summary of results: causes of piperacillin-tazobactam supply chain collapse.

**Figure 3.**
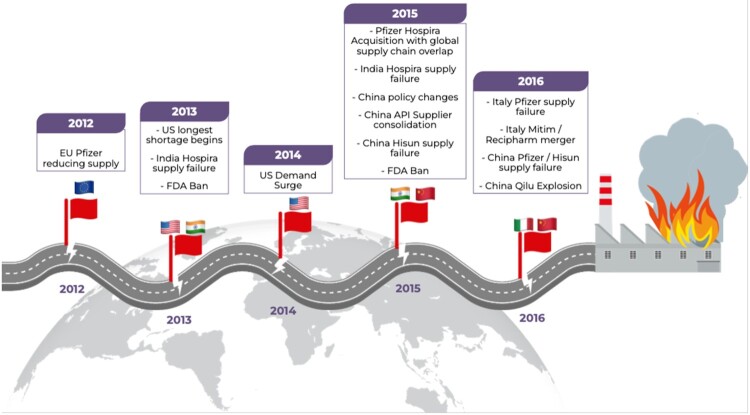
Red flags on the road to supply chain collapse.

In this case, supply chain management, which scholars described as a co-operative networked system (Mentzer et al., 2001), failed to co-operate (Figure 2). Our investigation demonstrates global capacity constraints in the Pfizer-led supply chain from 2012, and among Chinese suppliers from 2015. We identified demand increase coupled with supply constraints which emerged in the US, EU, UK, China, India and Italy. Capacity constraints emerged as Pfizer consolidated the acquired supply chains of Wyeth and Hospira, and sought cost benefits from outsourcing to China with Hisun. The delayed upgrade to Pfizer’s manufacturing in Italy proved ill-timed. The simultaneous impact of Chinese pharmaceutical policy, which supported domestic consolidation and global competitive advantage of their API suppliers, meant pip-tazo supply became concentrated at Qilu by 2016. During this time, a series of events occurred signalling increasing global supply chain risk (Figure 3). We interpret these signals as warning signs and call them ‘red flags’, which we explore below.

### Emerging ‘red flags’

#### Red flag 1: repeated manufacturing failures

We observed three locations experiencing supply disruption: China, India, and Italy. Causes were often manufacturing related, some leading to regulatory intervention to ensure product safety. Penicillin-based antibiotic API production, such as for pip-tazo, is based on sophisticated fermentation technology requiring dedicated sterile facilities (Elander, 2003). The need to properly ventilate and isolate manufacturing to reduce the risk of cross-contamination entails significant investment in dedicated production lines, and is a key rationale for seeking economies of scale at firm level (WHO, 2023). This complex process, and limitations of classical strain improvement and genetic engineering approaches to penicillin production (Sawant & Vamkudoth, 2022), explain the long-standing manufacturing challenges in this supply chain as noted in the reasons for US shortages (Table 1), leading some scholars to suggest a greater role for governments in antimicrobial manufacture (Shafiq et al., 2021). Indeed, Austrian government investment with Sandoz at Kundl is expected to boost resilient API production capacity through automation and modern technology (Sandoz, 2020). Despite biotechnological advances in production techniques, which have helped to minimise costs and improve yield efficiency, the need to reduce environmental damage from production waste remains challenging and costly (Haque et al., 2024). Consolidation makes economic sense for the companies involved in the supply chain. Our study confirms, however, that a smaller number of bigger plants entails higher risk of shortages if one plant fails. The lack of redundancy augments the risk of supply chain disruptions and associated deleterious impacts on public health (Gupta & Kayande, 2023).

#### Red flag 2: policy blindspot – undetected API supply concentration

Our study further suggests that Western policy makers did not see emerging API supply concentration because they had insufficient information to detect the early warning signs. During the competition enquiries related to the Hospira acquisition in 2015, they evoked alternative generic suppliers (Commerce Commission, New Zealand, 2015; European Commission, 2015). In the EU, for example, policy makers expected competitors to Pfizer to provide for patients stating ‘there are several suppliers that will remain on the market post-transaction’ (European Commission, 2015). However, they relied on assurances of alternative suppliers provided by ‘The Notifying Party’ i.e. Pfizer. While there were multiple theoretical suppliers, we have shown these firms themselves relied on increasingly concentrated API supply, evidenced in the US shortage reports and overlapping API supply from Hospira and Hisun to Pfizer. Such signals of growing risk in the global API supply chain went un-checked despite numerous ‘red flags’ (Figure 3). These warning signs are often difficult to detect (Faggioni et al., 2024). For example, when supply chain disruption leads to medicine shortages in Europe, there is no central mechanism for reporting (Bogaert et al., 2015; Miljković et al., 2022). Certification of Suitability (CEP), compliance with quality control of API coming into the EU, does not contain information about the location of API production (WHO, 2023). This lack of transparency on overlapping API supply locations and ‘volume dependency’ proved to be a key blindspot in pip-tazo’s case. Emerging supply risks remained undetected potentially inhibiting Western policy makers’ ability to set priorities for mitigations.

#### Red flag 3: Chinese pharmaceutical policy

Chinese policy making actively created comparative economic advantage and thereby incentivised domestic and international supply chain consolidation. Studying Chinese local production, WHO found ‘policy can be used to establish a pharmaceutical manufacturing sector capable of meeting the basic pharmaceutical needs of a national population given the necessary resources’ (WHO, 2017). Strong API capability was a cornerstone of China’s establishment of independent pharmaceutical production for domestic needs in the 1960s (European Parliament, 2023). Support for API production grew from natural ecological benefits, including climatic variety enabling the production of multiple raw ingredients, into structured market mechanisms. Comparative cost advantage was cemented with state subsidies; low interest rate loans from state banks; and less onerous environmental regulations related to chemical production (Lorin, 2020). In addition to lower input costs (electricity, coal, water, shipping), a World Bank study found that ‘if a typical Western API company has an average wage index of 100, the index is as low as 8 for a Chinese company’ (Bumpas & Betsch, 2009).

China’s National Development and Reform Commission said that APIs are ‘the long-term advantage of China’s pharmaceutical industry’ (Nikkei Asia, 2022). By 2017, the UK Medicines and Health Products Regulatory Authority estimated China was producing 40% of all APIs (Nikkei Asia, 2022), and by 2020, the EU was 85–90% dependent on the Chinese market for all ingredients (EU Dependence on the Chinese Pharmaceuticals Market, 2020). The prevailing Western discourse claims that regulatory and cost arbitrage allowed Chinese suppliers to dominate(WHO, 2023). Our study suggests that Pfizer’s cost rationalisation strategies also contributed to global API supplier consolidation as they too sought to benefit from this economic advantage by outsourcing to China.

### Co-ordination failures

A first co-ordination failure emerged in the Pfizer-led global supply chain as multiple simultaneous bottlenecks emerged (Pfizer in Italy, Hospira in India, and Hisun in China). Faggioni et al. (2024) point out that such interdependent global supply chains are vulnerable. Due to the wish to keep costs down, there is no backup mechanism generating an inherent tension between a desire for efficiency versus a need for resilience. The authors highlight that the lack of backup is unsuitable during crisis times and leads to major product shortages. These scholars have examined the role of collaborative networks and information sharing among supply chain partners as a strategy to improve resilience. They have suggested that resilience could be bolstered by developing dynamic capabilities to react before, during, and after disruptions (Faggioni et al., 2024). Our study demonstrates that in the case of pip-tazo, such strategies were lacking in the Pfizer-led supply chain leading up to 2016.

Our study suggests a second co-ordination failure. Local regulators appear to have assumed alternative sources existed to replace Pfizer’s reduced supply, while emerging concentration in the global supply chain seems to have gone undetected. From the analysis of competition enquiries we saw that firstly, most global markets did not have alternative local production (Commerce Commission, New Zealand, 2015). Secondly, EU regulators, for example, were reassured ‘that during a supply disruption of one of the main suppliers on the market, the other participants were able to supply sufficient volume to cover this shortage’ (European Commission, 2015). They expected competitors including Teva, Fresenius, Mylan, and Novartis/Sandoz to fill the gap. However, we did not find evidence that their enquiries analysed these competitor firms’ overlapping API supply locations or volume dependency. Indeed, potential alternative Italian supplier Mitim had announced plans for a new beta-lactam production line, but was being acquired by Recipharm in 2016. Fresenius Kabi was awaiting approval of its generic formulation, and only planned to use the new Mitim/Recipharm facilities for API production from 2017 (K. Macdonald, 2017; Palmer, 2016).

This second co-ordination failure therefore presents a possible policy gap. In some jurisdictions, policy makers impose an obligation to report impending supply constraints into individual markets, but there is no collective oversight across geographies (Årdal et al., 2021). Simultaneous consolidation of Pfizer-led and Chinese suppliers was not detected, with actors, whether public or private, only focussed on their part of the chain. Neither the firms involved, nor policy makers, had responsibility for supply chain resilience. Our results further indicate that this risky two-pronged pressure on pip-tazo supply went undetected because policy makers focussed on local signals and national-level risks. They did not appear to have sufficient information to generate an overview of the global supply chain***,*** rendering them unable to evaluate the interlinkages. This blindspot, i.e. missing scrutiny of interlinkages, is reflected in analysis of SCR in the pharmaceutical sector (Faggioni et al., 2023). To our knowledge this policy gap has not been mitigated since the period under study. Current policy debates on security of supply similarly often focus at national or at best regional level (Shafiq et al., 2021). These often focus on the potential for re-building local manufacturing capacity (re-shoring) (Shore et al., 2022). Our study suggests local solutions may not be enough to re-build resilience in antibiotic supply.

### Reshoring: a dose of reality

Indeed, attitudes to SCR are changing, particularly since medicine supply chain collapse experienced during the Covid-19 pandemic (Liza et al., 2023) (Faggioni et al., 2024). Export bans and protectionism re-emerged in so-called ‘Sicken Thy Neighbour’ attitudes which caused policy makers and companies to rethink geographic distribution of global supply chains (Evenett, 2020). Building local production, i.e. re-shoring, has therefore become a policy concern, and proposed response to SCR across economic contexts (Khan & Rauf, 2024). India, dependent on China for many APIs for its generic pharmaceutical industry (Bjerke, 2022), implemented new policies to encourage domestic production of bulk pharmaceutical products. These include a $900 m Production Linked Incentive specifically for 53 important APIs and other intermediate products for which India is highly dependent on China (Nikkei Asia, 2022). The US Biomedical Advanced Research and Development Authority (BARDA) began to stockpile critical medicines, including pip-tazo, under the Strategic Active Pharmaceutical Ingredient Reserve (SAPIR) amidst calls for US government subsidies for investment in new domestic manufacturing (Edwards, 2021). In a rethink of European government and private sector co-operation, in 2020, the Austrian government supported a €150 m joint investment in the Sandoz Kundl manufacturing site ‘to strengthen the long-term future of integrated antibiotics manufacturing in Europe’ (Sandoz, 2020). The French government has recently invested in reshoring to support locally produced paracetamol API supply. This case is instructive. At the outset, the CEO of Seqens, the French API producer said: ‘We are a private company so we can’t go ahead if there is no business case … without the support of states, such reshoring will not happen’ (Abboud & Peel, 2020). Herein lies the crux of the challenge for Western policy makers. Shortages are directly or indirectly driven by the economic viability of supply chains which often rely on single source ingredients (Pandey et al., 2024).

### Market dynamics

In financialised economies, the private sector will not invest in antibiotic API capacity without state subsidies under current market conditions. Western governments acknowledge current antibiotic market dynamics whereby pressure on finished medicines prices flows into pricing pressure on API input materials, and contributed to relocation of production to lower cost countries in Asia (Oehler & Gompf, 2020). Indeed, French and German government studies found that European antibiotic markets, as they are structured today, cannot support the price increases necessary to rebuild security of API supply through reshoring (Roland Berger, 2018; WHO, 2023). A study in the US questioned whether shortages are being driven by some drug prices being set too low (Hernandez et al., 2020). Pricing being ‘too cheap’ has been shown to deter investment, e.g. UK tender rules are resulting in companies pulling out of supplying into the medicines pricing scheme (Fick & Grover, 2023). For pip-tazo, the EU Pfizer/Hospira competition enquiry noted the generic sterile injectables market is primarily driven by tenders with price an important supplier selection criterion (European Commission, 2015). Generic use has been increasing in Western countries as health systems seek to manage costs (Greene, 2014). IQVIA data shows generics were 50% of prescriptions in US in 2005 but 90% by 2020 (Nikkei Asia, 2022). However, in an interesting contribution from legal scholars, Hemel and Ouellette analyse what they term the trilemma of the need for off-patent drug policy to accommodate price, quantity and quality but point out that typically policies which facilitate one or two of those outcomes satisfactorily are forced to compromise on the third. The economics of this transition in health systems often entails a race to the bottom on price throughout the value chain risking reduced manufacturing capacity, quality concerns, and eventually global shortages (Hemel & Ouellette, 2022).

Contributing to the knowledge gap on non-Western antibiotic industries, and emerging scholarship on antibiotic geographies (Bjerke, 2022), however, our study shows that contrary to the prevailing Western view which ‘blames’ Chinese (and Indian) manufacturers for exerting pricing pressure, ‘blame’ for policy constraints can also be apportioned in Western regulatory jurisdictions. In both the West and China, manufacturing standards, state aid rules, environmental protections, and pricing of generics, often driven by mandatory procurement tenders, have all rendered antibiotic markets less commercially attractive. Emerging scholarship suggests the Chinese API producers, on which the world now relies, are under economic pressures similar to those which incentivised past capacity reductions in the West and China is also experiencing critical antibiotic shortages (Zhang, 2023). Indeed, these worsening economic realities of global antibiotic markets pose new risks for antibiotic API supply. Reshoring advocates are thus faced with a costly reality reflecting the meso-analysis conducted in Sweden by Baraldi et al. (2023) demonstrating that reshoring strategies entail trade-offs with pros and cons for different stakeholders (Baraldi et al., 2023). In order to counteract this trajectory, while adhering to environmental and sustainability objectives, Western governments would need to subsidise reshoring both on the capital investment side and on the antibiotic purchasing side.

### Western and Chinese policy contexts

#### Pfizer’s financialised context

**Figure 4.**
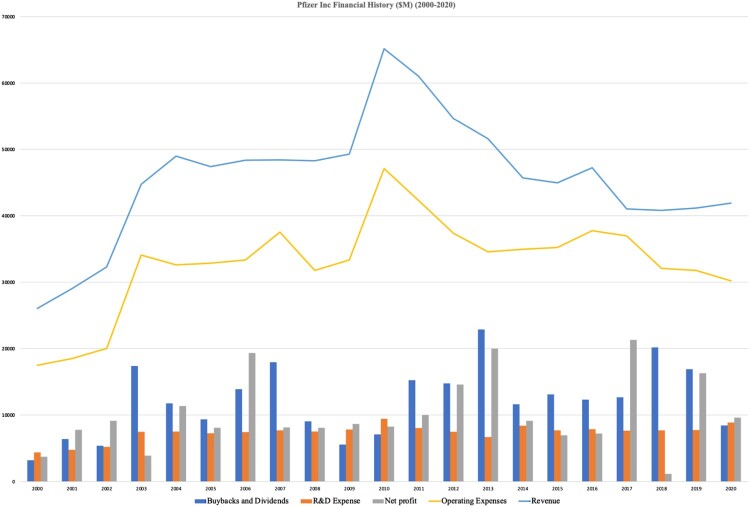
Pfizer’s financial history (2000–2020). Source: Pfizer Inc (Data extracted from Datastream, access provided by Geneva Graduate Institute June 15, 2021).

**Figure 5.**
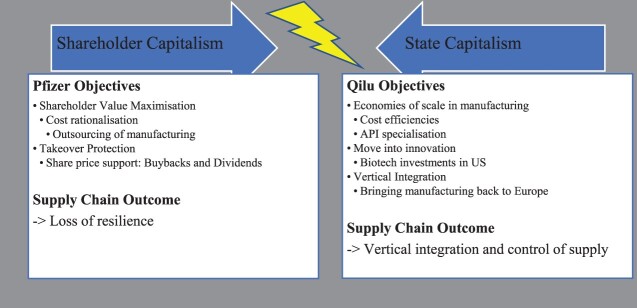
Forms of capitalism collide: supply chain outcomes.

Responding to calls for additional research on industrial policy and comparative political economy (Bulfone, 2023), and more broadly to literature on financialisation of the pharmaceutical industry (Lazonick et al., 2017, 2019), our study has shown that the contrasting national policy environments of Pfizer and Qilu both impacted SCR (Figure 5). Pfizer’s supply chain decisions were made in the context of the financialised political economy in which the firm operates. Scholars of financialisation have analysed Western firms governed by shareholder capitalism striving to maximise shareholder value (Lazonick, 2010). One mechanism deployed to support share prices is share buybacks, funded by cost cutting, including by outsourcing global supply chains (Lazonick, 2011). Pfizer’s financial journey, including seeking lower cost API production in China, epitomises this trajectory (Gibson, 2018). From 2011 to 2016, share price appreciation was supported by giving $90bn back to shareholders, despite losing revenues of $23bn in patent expiries (Pfizer Inc, 2016) (Figure 4). However, our study has shown that contrary to critical literature which describes firms such as Pfizer as disinterested in investment in low-value medicines such as generic antibiotics, Pfizer did in fact continue to invest (Busfield, 2020). The manufacturing upgrade in Italy, and the joint venture in China were planned to support the API supply chain. However, in this case, planned investment in outsourced capacity with Hisun failed to materialise in time, and was therefore unavailable to mitigate emerging capacity constraints elsewhere (Liu, 2017).

#### State capitalism: international trade objectives

In contrast, China’s socialist market economy, often described in the West as state capitalism, has included international trade objectives (Fotin, 2023), which the US Senate described as structured ‘to make China’s domestic industry the medicine cabinet of the world’(Oehler & Gompf, 2020). Indeed, in expounding the role that pharmacists play in ensuring access to quality medicines, an expert pointed out that ‘if China stopped supplying ingredients, pharmacy shelves [in the US] would be empty within months’ (Gibson, 2020). Scholars have demonstrated the increasing role China has played in global antibiotic trade, including the important API supply route to the Indian generic pharmaceutical industry (Zhang & Bjerke, 2023), which in turn supplies 20% of global generics (40% of US demand and 25% of EU demand) (Guerin et al., 2020). In antibiotic markets Chinese firms have therefore achieved these trade policy goals (Y. Bao et al., 2021). Indeed, by 2023, Qilu described itself as ‘the world’s major supplier of cephalosporines, tazobactam, amikacin sulphate and carbapenems for over a decade’ (Qilu Pharmaceutical, 2023). However, the Chinese company is now present globally, throughout the pharmaceutical value chain. Qilu recently rebuilt lyophilised cake manufacture in the EU, reshoring capabilities previously moved to China (Invest in Spain, 2021). In the US, Qilu established the first Chinese-owned, US-based, biotech incubator in 2017 (Weisman, 2017).

## Implications for pharmaceutical policy

Antibiotic shortages have become a global concern highlighting fundamental failings in the pharmaceutical production landscape (Sharland et al., 2024; Skender & Zhang, 2024). The global policy space is incoherent, with downstream access consequences as stakeholders juggle national interests, nationalist sentiment, and public health against a backdrop of corporate or state interests depending on the domestic political economic context (Zhang & Bjerke, 2023). The ‘red flags’ identified in our study therefore suggest a role for policy makers in global supply chain risk surveillance. Had policy makers uncovered emerging global risk, rather than local, could this supply chain ‘accident’ have been avoided? Had data been shared across geographies, could mitigations have been deployed in time? Do we therefore lack a policy framework to signal emerging global supply risks?

Our findings suggest several policy gaps which impacted on the lack of supply chain resilience. Firstly, policy makers lacked visibility on ‘volume dependency’ and therefore missed signals on building concentration in the pip-tazo supply chain. This suggests bottlenecks could be better pre-empted with mandatory disclosure of degree of dependency on API suppliers by market authorisation holders. International co-ordination of such data could provide early warning signals of emerging concentration risks across global supply chains. Secondly, taking a global view of supply chain risk would allow priority setting for any new investments in re-shoring. Such strategies will be expensive to implement, and they risk being inefficiently deployed without international co-ordination. With data sharing, priority could be given to the most vulnerable areas where global API supply currently relies on few, or single plants. Thirdly, we have shown that the economics of antibiotic API manufacturing are also impacted by a number of non-pharmaceutical policy decisions (e.g. state aid, environmental standards, and procurement rules) suggesting a whole-of-government approach to supply chain policy setting could be beneficial. Our findings confirm suggestions from previous studies that international data sharing would be beneficial considering the global shortage effects which can emerge from a single point of failure (Årdal et al., 2021; Pandey et al., 2024). However, acknowledging the inbuilt opacity in global supply chains, our study also highlights the complexity of implementing cross-border data exchange. A possible starting point therefore could be to link supply resilience monitoring to the WHO priority pathogen list (WHO, 2024) to enable focus on the most critical antibiotic shortage risks.

### Limitations and future research

In terms of future research, a better understanding of the impact on patient populations and healthcare providers, who are in the front-line of managing shortages day to day, would be helpful (Said et al., 2018). While studies exist in community settings, we found fewer qualitative studies involving hospitalised patient perspectives. A recent study from Hungary on managing shortages in inpatient care has shown the role that good communication and collaboration between doctors and pharmacists as well as robust information sources played in ensuring appropriate antibiotic treatment for patients (Lőrinczy et al., 2023). In addition, a study from the field of oncology serves as a potential model for future qualitative studies with patients in order to understand their awareness and experience of shortages during treatment (Hantel et al., 2020).

Future case studies of specific antibiotic shortages would also be beneficial in order to provide additional empirical evidence of specific causes and inform potential mitigations. Notwithstanding the methodological mitigations outlined, our study retains several limitations due to the lack of disclosure of API locations by market participants. Increasing sensitivity around global supply chains in both the West and China emerged during the period of our study. We found local Chinese data sources became increasingly hard to access due to effects of the pandemic. This therefore precluded our ability to cross-check data sources between geographies and forced us to rely on publicly available data to reconstruct the pip-tazo supply chain. Further studies of individual antibiotic trajectories would benefit from the ability cross reference insider recollections of key inflection points with findings from available public data.

In terms of prioritisation, shortages of penicillins have been reported globally over a long-time frame (Harbarth, 2000; Nurse-Findlay et al., 2017; Wyber et al., 2015). Recently, global shortages of amoxicillin, particularly impacting paediatric populations, have been of particular concern (Cohen et al., 2023). Such shortages have highlighted the policy gaps outlined in our paper. A recent commentary timed with the UN High Level Meeting on AMR in September 2024 was entitled ‘Recent amoxicillin global shortages mask a wider policy failure’ (Sharland et al., 2024). The immediate shortage cause has been ascribed to increased demand post-pandemic due to resurgence of respiratory and streptococcal infections, but our study suggests a methodology which explores the SCR trifecta of demand, supply and co-ordination, as we have done for pip-tazo, may support initiatives to generate sustainable access to critical ‘key access’ antibiotics such as these.

## Conclusion

Policy makers remain ill-equipped as they explore medical supply chain risk mitigations. They lack visibility on interlinkages across global footprints and have insufficient data on ‘volume dependency’ of market authorisation holders. Despite multiple examples of market failure, which inhibit access to low profitability medicines, Western policy making still assumes ‘the market’ will fill supply gaps. Our study demonstrates that such assumptions proved false. In our cautionary tale, pip-tazo went from ‘blockbuster’ drug (defined as $1bn in annual revenues) to global shortage in just 6 years despite multiple ‘red flags’ signalling increasingly risky supply chain concentration.

A combination of pharmaceutical and non-pharmaceutical policies pushed Western firms to exit manufacturing of critical antibiotics while Chinese policy supported the growing global dominance of Chinese API suppliers. However, recent studies suggesting worsening economics of antibiotic API production in China mean the outlook for supply chain resilience appears bleak. Our findings support the need for a new relationship between the state and pharmaceutical firms to govern sustainable access to less commercially attractive medicines like generic antibiotics where economic pressures contribute to supply chain vulnerability. A policy framework to monitor global supply chains for critical essential medicines through international data sharing and co-ordination of risk mitigation efforts may facilitate re-building resilience.

## Data Availability

All data generated or analysed during this study are included in this published article.

## Footnotes

1 Competition enquiries are undertaken by regulatory authorities who look into risks of distortion of competition due to market dominance or anti-competitive behaviour by firms. In this case, the market enquiries related to risks of excessive market share in some jurisdictions when Pfizer acquired Hospira in 2015 as both firms were major suppliers of piperacillin-tazobactam.
